# Observation of acoustic Dirac-like cone and double zero refractive index

**DOI:** 10.1038/ncomms14871

**Published:** 2017-03-20

**Authors:** Marc Dubois, Chengzhi Shi, Xuefeng Zhu, Yuan Wang, Xiang Zhang

**Affiliations:** 1 NSF Nano-scale Science and Engineering Centre (NSEC), University of California, Berkeley, 3112 Etcheverry Hall, Berkeley, California 94720, USA; 2 Materials Science Division, Lawrence Berkeley National Laboratory, 1 Cyclotron Road, Berkeley, California 94720, USA

## Abstract

Zero index materials where sound propagates without phase variation, holds a great potential for wavefront and dispersion engineering. Recently explored electromagnetic double zero index metamaterials consist of periodic scatterers whose refractive index is significantly larger than that of the surrounding medium. This requirement is fundamentally challenging for airborne acoustics because the sound speed (inversely proportional to the refractive index) in air is among the slowest. Here, we report the first experimental realization of an impedance matched acoustic double zero refractive index metamaterial induced by a Dirac-like cone at the Brillouin zone centre. This is achieved in a two-dimensional waveguide with periodically varying air channel that modulates the effective phase velocity of a high-order waveguide mode. Using such a zero-index medium, we demonstrated acoustic wave collimation emitted from a point source. For the first time, we experimentally confirm the existence of the Dirac-like cone at the Brillouin zone centre.

## Abstract (web summary)

Impedance mismatch between acoustic metamaterials and a surrounding medium hinders efficient applications, especially for zero-index materials. Here, Dubois *et al*. utilize the Dirac-like dispersion in a double-zero-index material to overcome this problem and to collimate sound.

Acoustic wave behaviour is affected by material parameters such as mass density and bulk modulus of the media where the wave propagates. Metamaterials provide a new strategy to design unprecedented material properties that do not exist in nature^1^,^2^,^3^,^4^,^5^,^6^,^7^,^8^,^9^,^10^,^11^,^12^,^13^,^14^,^15^,^16^,^17^. Metamaterials with negative effective mass density and bulk modulus were experimentally developed using locally resonant sonic crystal^1^ and Helmholtz resonators^2^, respectively. These materials are called single negative materials as only one of their parameters is negative. The refractive index of such materials is dominantly imaginary, due to a band gap. With proper design of the multiple scattering in bi-periodic crystals, single negative materials can be used to realize a superlens that breaks the diffraction limit^3^. Double negative materials with negative refractive index whose mass density and bulk modulus are simultaneously negative were demonstrated using a one-dimensional waveguide with membranes and side holes^5^,^18^ and coiled space structures^19^,^20^,^21^. Anisotropic materials were realized for the design of an acoustic hyperlens^6^, super-resolution imaging^7^ and cloaking^8^,^9^. While single zero materials have been explored both in electromagnetic (epsilon near zero)^22^,^23^,^24^,^25^ and acoustic metamaterials (density near zero)^26^,^27^,^28^,^29^, these media suffer from low-transmission due to an impedance mismatch. In acoustics, the impedance of a material is given by 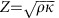, where *ρ* is the mass density and *κ* is the bulk modulus. An acoustic double zero refractive index metamaterial with simultaneously zero density and infinite bulk modulus achieving finite impedance overcomes such an obstacle (Supplementary Notes 1 and 2; Supplementary Figs 1 and 2). Recently developed electromagnetic metamaterials with a Dirac-like cone at the Brillouin zone centre exhibit double zero-index properties^30^,^31^,^32^,^33^. These electromagnetic metamaterials consist of periodic scatterers with phase velocity lower than the surrounding materials^30^,^31^. But this requirement is extremely challenging for airborne sound applications because the sound speed in air is slow compared with other materials.

Here, we apply cylindrical scatterers with height larger than the background air channel in a two-dimensional waveguide, where the acoustic phase velocity is smaller than air sound speed for a high-order waveguide mode to realize an acoustic double zero refractive index metamaterial. The acoustic double zero refractive index metamaterial is used to collimate cylindrical waves emitted from a point source at the centre of the medium. Our analysis of the collimated acoustic beam exhibits a high-directivity performance. The holey structure of the double zero index metamaterial allows us to measure the acoustic field inside the medium and map the reciprocal space to confirm the existence of a Dirac-like cone at the Brillouin zone centre experimentally. Acoustic zero refractive index metamaterials open new possibilities for effective acoustic wave engineering in applications such as ultrasound medical imaging and underwater communication.

## Results

### Design of an acoustic double zero index metamaterial

**Figure 1:**
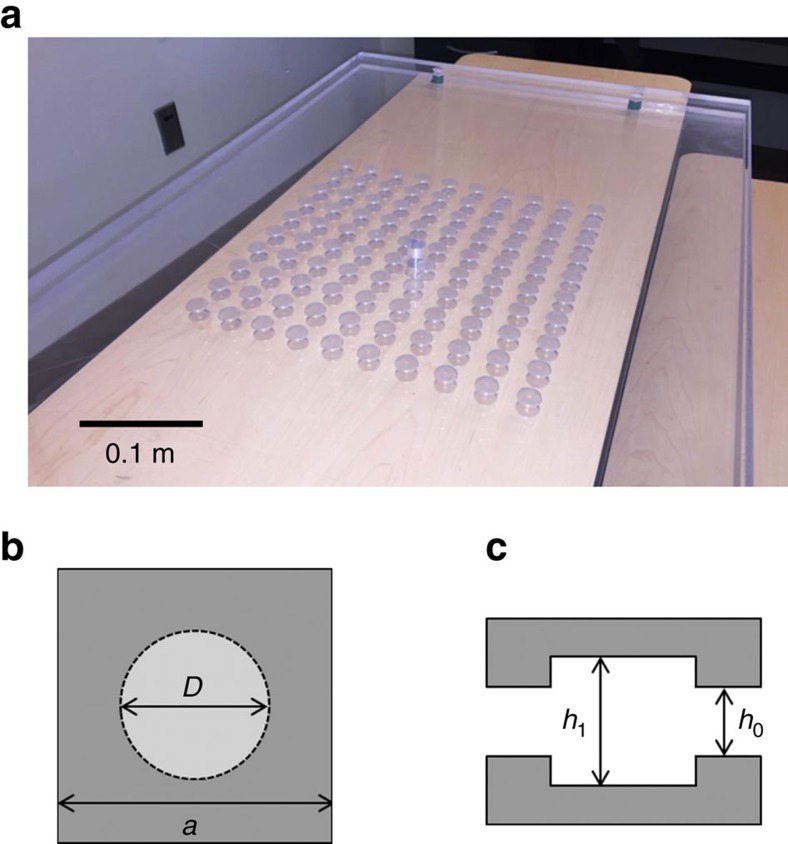
Acoustic metamaterial with simultaneous zero effective mass density and infinite effective bulk modulus. (**a**) Photograph of the fabricated sample with square lattice of 10 × 10 symmetric blind holes constituting an array of cylindrical scatterers. A through hole provides access to the centre of zero index metamaterial for a point source to excite the first order waveguide mode. Four spacers located at the corners of the sample ensure the height of the air channel in the waveguide and the alignments of the top and bottom plates. (**b**,**c**) Top and side views of a unit cell of the zero refractive index metamaterial, respectively. Grey areas mark the solid structures of the waveguide. The light grey circle in the top view denotes the blind holes on the top and bottom plates inside the waveguide. The top and bottom plates are symmetric about the central plane. *D*=16 mm, *h*_0_=10 mm, *h*_1_=14.5 mm and *a*=30 mm.

**Figure 2:**
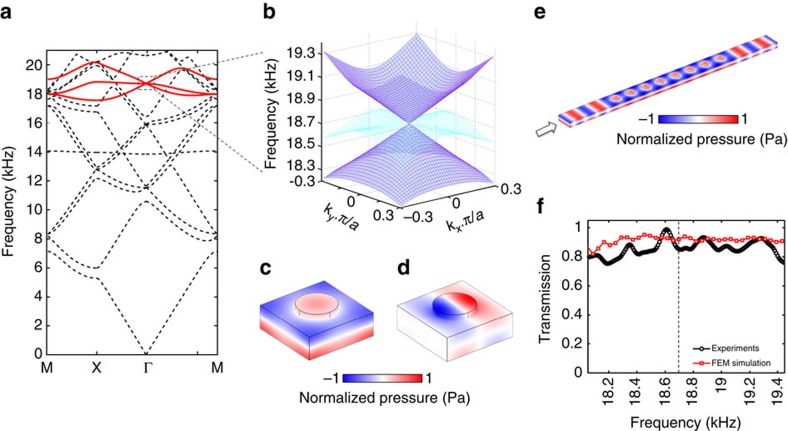
Dirac-like cone dispersion of designed double zero index metamaterial. (**a**) Calculated band structure of the zero index metamaterial. A Dirac-like cone is located at Brilliouin zone centre for the first order waveguide mode at 18.7 kHz. It is formed by the three bands highlighted with red curves. These three bands are degenerate at the Dirac point. (**b**) Zoom in of three-dimensional dispersion surfaces near the Dirac-like cone. The dispersion surfaces below and above the Dirac point (blue) have negative and positive group velocity, respectively. The metamaterial has zero refractive index at the Dirac point. The flat band (cyan) is not excited in our experiment due to symmetry. (**c**,**d**) calculated pressure fields of the first order waveguide mode showing degenerate monopolar and dipolar behaviours at the Dirac point, respectively. The fields are anti-symmetric along the out-of-plane direction, as expected for the first order waveguide mode. Monopolar and dipolar behaviours modulate the bulk modulus and density of the material, respectively. These modulations enable simultaneous zero effective density and infinite effective bulk modulus at the Dirac point. (**e**) Full-wave simulation of a plane wave propagating through eight unit cells at 18.7 kHz. The solid side walls are equivalent to periodic boundary conditions forming a two-dimensional square lattice. (**f**) Corresponding simulated (red) and measured (black) normalized transmissions between 18 and 19.4 kHz. The simulated transmission including standard viscous loss over this frequency band is 91%, and the measured transmission is 86%. The absence of a band gap and near total transmission demonstrates impedance matching between the double zero index metamaterial and surrounding waveguide.

In this letter, we experimentally realize an acoustic metamaterial with simultaneous zero mass density and infinite bulk modulus induced by a Dirac-like cone at the Brillouin zone centre by periodically varying the thickness of an air channel in a two-dimensional waveguide (Fig. 1), resulting in the change of the effective sound speed of the first order waveguide mode (Supplementary Note 3; Supplementary Fig. 3)


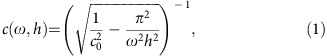


where *c*_0_=343 ms^−1^ is the sound speed in air, *ω* is the angular frequency and *h* is the thickness of the air channel. The periodic cylindrical air columns with larger air thickness in Fig. 1, have slower phase velocity than in the surrounding waveguide. With proper scatterer dimensions and lattice constant, this material exhibits a Dirac-like cone at the Brillouin zone centre for the first order waveguide mode (Fig. 2a,b). This Dirac-like cone is formed by the degeneracy of a monopolar mode and two dipolar modes (Fig. 2c,d). The monopolar mode modulates the effective bulk modulus and the dipolar mode affects the effective mass density^34^. The pressure field of the other dipolar mode is orthogonal to the one shown and is not excited. The lowest band of the Dirac-like cone has a negative group velocity as the wave vector decreases with increasing frequency (Fig. 2a,b). Consequently, the material behaves as a double negative medium; similarly for double positive behaviour for the upper cone. At the Dirac point, where these two bands are degenerate, the effective mass density and the inverse of the bulk modulus are simultaneously zero. This gapless band structure allows high-transmission because the acoustic impedance remains finite. A first order waveguide mode plane wave is generated and propagates through a slab of eight unit cells in order to test the transmission at normal incidence (Fig. 2e). The measured transmitted amplitude is nearly constant across the Dirac-like cone (Fig. 2f). This result indicates the absence of a band gap in the fabricated metamaterial. Furthermore, the measured 86% amplitude transmission confirms impedance matching between our designed metamaterial and the surrounding medium. One-dimensional conical dispersion was observed in coiled space structures^27^,^28^. However, the narrow channels in the complex structure increase the viscosity loss^35^. Thus, it is difficult to achieve double zero index under such a high-loss and perfect transmission can hardly be observed.

### Acoustic collimation with the double zero index metamaterial

**Figure 3:**
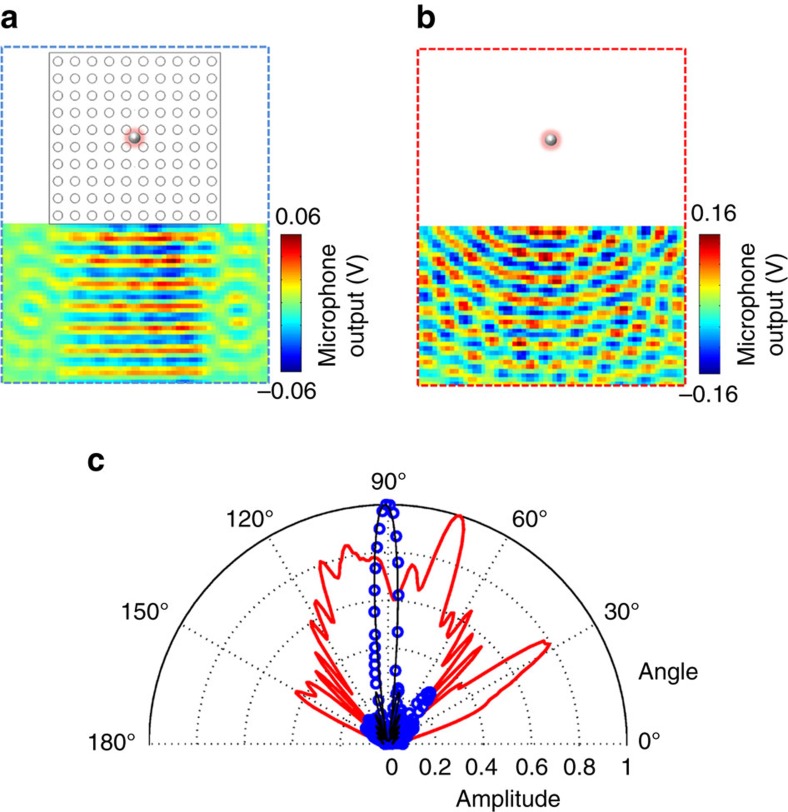
Acoustic plane wave generation from a point source in the zero refractive index metamaterial. (**a**) Measured pressure field radiated by acoustic point source embedded in zero refractive index metamaterial. The zero refractive index metamaterial collimates the emission from a point source to a plane wave. (**b**) Measured pressure field radiated by an acoustic point source in the empty waveguide. Without zero refractive index material, a cylindrical wave pattern is obtained in the waveguide. (**c**) Directivity of a collimated plane wave. The blue circles and red curve are directivities corresponding to the pressure field radiations shown in **a**,**b**, respectively. The black curve is the calculated directivity of an ideal line source with the same size (10 unit cells, that is, 6.5 wavelengths). The zero index metamaterial collimates the acoustic pressure field within 11±1°, close to 10.6° given by the theoretical limit of the line source. The noise observed on both experimental results is due to the spurious reflection from the waveguide edges.

Acoustic wavefront engineering and plane wave generation is crucial for the applications such as imaging and sensing^36^. Traditional acoustic plane wave generation requires implementation of large arrays of sources together with complex controlling circuits. Indeed, single-acoustic sources are small compared with wavelength, emitting cylindrical waves in two-dimensional space whose intensity decays along the direction the wave travels in. As the phase velocity is infinite in a zero index material, the phase of the acoustic field is uniform throughout the material. Therefore, one can design the wavefront of the outgoing field by shaping the interface between the zero index material and the surrounding medium^37^,^38^. In our experiment, an acoustic point source exclusively exciting the first order waveguide mode is placed at the centre of a square metamaterial sample consisting of 10 by 10 unit cells (Fig. 1a). The pressure field radiated by the point source at the Dirac point frequency (18.7 kHz) is scanned, and shown in Fig. 3a,b with and without the metamaterial, respectively. We observe that the phase along the edge of the metamaterial is uniform. Therefore, the cylindrical wavefront emitted by the point source is collimated into a plane wave. This experimental result confirms that the metamaterial acts as a double zero index medium at this frequency. The amplitude of the collimated plane wave is confined within 11±1° (Fig. 3c). This directivity performance is near the theoretical limit (10.6°) obtained from a line source with the same dimensions (Supplementary Note 4; Supplementary Fig. 4). The measured amplitude of the collimated plane wave contains about 30% of the point source emission. The reduced transmission has already been reported and explained in optics and is due to the low-available density of states^32^.

### Observation of a Dirac-like cone at the Brillouin zone centre

**Figure 4:**
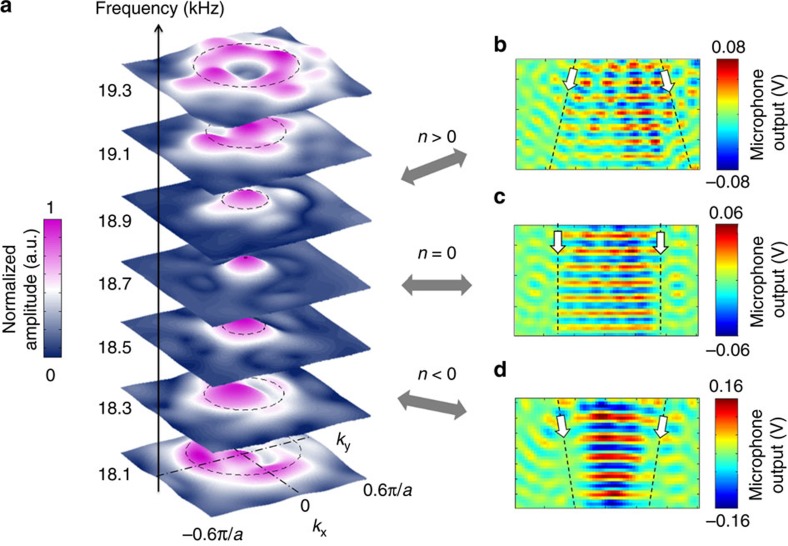
Measurement of the Dirac-like cone and the associated interface acoustic refraction. (**a**) Experimentally resolved reciprocal space of the zero index metamaterial near the Brillouin zone centre at different frequencies. A ring is observed at low-frequencies and shrinks to a point at 18.7 kHz. The ring opens again as the frequency increases. The black dashed circles represent the calculated equifrequency contours of the Dirac-like cone shown in Fig. 2b and agree well with the measurement. (**b**–**d**) Measured pressure fields outside the metamaterial at 19.1 kHz, 18.7 kHz and 17.9 kHz, respectively. The black dashed lines and white arrows indicate the acoustic beam profiles and propagation directions. A focusing pattern is observed at 17.9 kHz due to the double negative index property. An acoustic plane wave is obtained at 18.7 kHz as a result of the double zero index metamaterial. The acoustic beam expands, when propagating at 19.1 kHz because the material parameters are both positive.

The existence of a Dirac-like cone at the Brillouin zone centre provides a key evidence of double zero index property^30^,^31^,^32^,^33^. As the metamaterial structure is hollow, one can directly access the field within and characterize the equifrequency contour in reciprocal space. The pressure field inside the metamaterial is scanned for different frequencies and mapped onto reciprocal space with a two-dimensional Fourier transform (Fig. 4a). A Dirac-like cone is observed at the Brillouin zone centre between 18.1 and 19.3 kHz with its Dirac point at 18.7 kHz. This measured Dirac-like cone matches the theoretical dispersion shown in Fig. 2a,b. At the Dirac point, the medium exhibits double zero index property. Subsequently, we measure a collimated plane wave at 18.7 kHz (Fig. 4c). The upper band of the Dirac-like cone gives a positive group velocity for an acoustic wave propagating in the metamaterial. Therefore, a refraction effect occurs at the interface resulting in the expansion of the acoustic beam observed at 19.1 kHz (Fig. 4b). For frequencies below the Dirac point, the metamaterial acts as a double negative medium, while the outer waveguide is a double positive medium. They form a negative–positive interface and focus the acoustic beam (Fig. 4d). The measured amplitude of the focusing beam compared with the point source emission at the same frequency is 60%. This continuous evolution of refractive index from double negative to double positive is also confirmed by the measured acoustic field inside the metamaterial. At 18.3 kHz, the acoustic field envelope merges into the point source due to the double negative refractive index (Supplementary Movie 1). The acoustic field envelope propagates away from the point source at 19.1 kHz where the refractive index is double positive (Supplementary Movie 3). At 18.7 kHz, the excited acoustic field envelope is breathing with a uniform phase distribution because of the double zero refractive index (Supplementary Movie 2).

## Discussion

In conclusion, we have experimentally demonstrated the first acoustic double zero index metamaterial. The measurement of the reciprocal space inside the metamaterial reveals the presence of a Dirac-like cone at the Brillouin zone centre. As a consequence, this metamaterial possesses simultaneous zero effective mass density and infinite bulk modulus at the Dirac point. This allows impedance matching with a background medium and is confirmed by a transmission measurement. Acoustic beam collimation from a point source is also demonstrated using the zero index property in this passive structure. Acoustic zero index materials offer unique wavefront engineering, providing a novel platform for the exploration of fundamental physics such as transformation acoustics, sound dispersion and phase matching.

## Methods

### Experimental setup

The band structure of our designed material is calculated using a commercial finite element solver. This designed material contains cylindrical air scatterers with radius 8 mm and height 14.5 mm forming a square lattice with lattice constant 30 mm in a two-dimensional 10 mm thick air waveguide (Fig. 1). This waveguide is formed by two acrylic plates over a 930 mm by 540 mm area. The blind holes forming the 10 by 10 cylindrical scatterers are drilled by a CNC machine. Spacers are placed at the four corners of the waveguide to form the 10 mm thick air channel between the two acrylic plates. The waveguide with eight unit cells used to measure transmission shown in Fig. 2f is also fabricated by the CNC machine. Two PUI AST-1532MR-R speakers generating airborne sound with opposite phase are assembled face to face at the top and bottom plates. They form an acoustic point source exclusively exciting the first order waveguide mode at the centre of our 10 by 10 unit cell sample. The acoustic signal is measured by a CUI CME-1538-100LB microphone and amplified by Reson VP2000 voltage preamplifier EC6081. A Stanford research systems SR830 lock-in amplifier is used to read the amplitude and phase of the acoustic signal. Two VELMEX MN10-0150-E01 motors controlled by VXM stepping motor controller are used to move the microphone and scan the acoustic field in the waveguide with a scan of 75 by 60 points and step size 7.6 mm.

### Data availability

The authors declare that all data supporting the findings of this study are available within the paper and its Supplementary Information files.

## Additional information

**How to cite this article:** Dubois, M. *et al*. Observation of acoustic Dirac-like cone and double zero refractive index. *Nat. Commun.* **8,** 14871 doi: 10.1038/ncomms14871 (2017).

**Publisher's note**: Springer Nature remains neutral with regard to jurisdictional claims in published maps and institutional affiliations.

## Supplementary Material

Supplementary Information Supplementary Figures, Supplementary Notes and Supplementary References

Supplementary Movie 1 The acoustic field envelope merges into the point source at 18.3 kHz because of the double negative refractive index below the Dirac-like point

Supplementary Movie 2 The acoustic field envelope is breathing with a uniform phase distribution at 18.7 kHz because of the double zero refractive index at the Dirac-like point.

Supplementary Movie 3 The acoustic field envelope propagates away from the point source at 19.1 kHz because of the double positive refractive index above the Dirac-like point.

Peer Review File
