# Supplementary material for: Observation of acoustic Dirac-like cone and double zero refractive index
Source: Nat Commun. 2017 Mar 20;8:14871. doi: 10.1038/ncomms14871 (PMC5364384; doi:10.1038/ncomms14871)
Supplement: Supplementary Information — Supplementary Figures, Supplementary Notes and Supplementary References [file ncomms14871-s1.pdf]

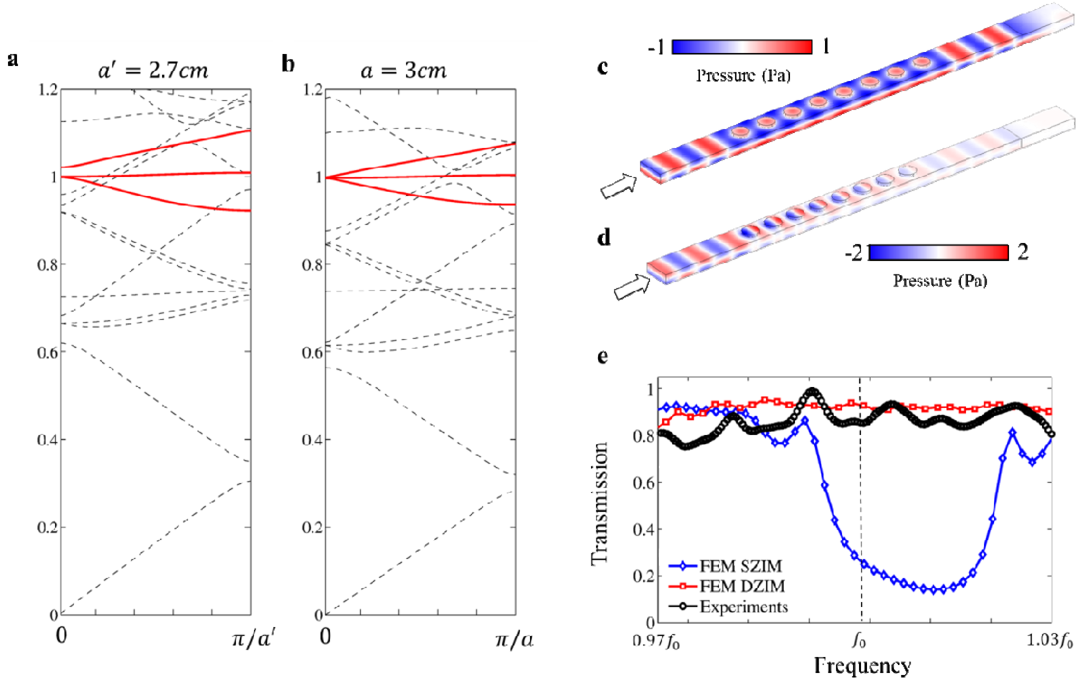

Supplementary Figure 1: Comparison of single and double zero index metamaterials. (a, b) Dispersion relations of one-dimensional arrays of cylindrical scatterers with lattice constant 2.7 cm and 3 cm, respectively. The frequencies are normalized with the zero index frequency f_0 of the associated structure. For lattice constant 3 cm, the same as the structure in the main text, a Dirac cone is observed, giving double zero index properties. The three bands forming this Dirac cone are highlighted with red curves. Once the lattice constant is detuned to 2.7 cm, the Dirac cone opens up to a bandgap, resulting in single zero index properties. (c, d) Full wave simulation of transmissions of one-dimensional double and single zero index metamaterials with eight unit cells for normal incidence, respectively. High transmission can be observe for double zero index metamaterial. A large portion of acoustic energy is reflected by the single zero index metamaterial, resulting in a low transmission through the medium. (e) Simulated (red) and measured (black) transmission through the double zero index metamaterial and simulated transmission through the single zero index metamaterial (blue) between 0.97 f_0 and 1.03 f_0 for normal incidence. The simulated and measured transmissions of the double zero index metamaterial are 91% and 86%, respectively. This high transmission is a result of impedance matching between our designed double zero index metamaterial and the surrounding waveguide. Due to impedance mismatch, only 30% transmission can be observed for the single zero index metamaterial.

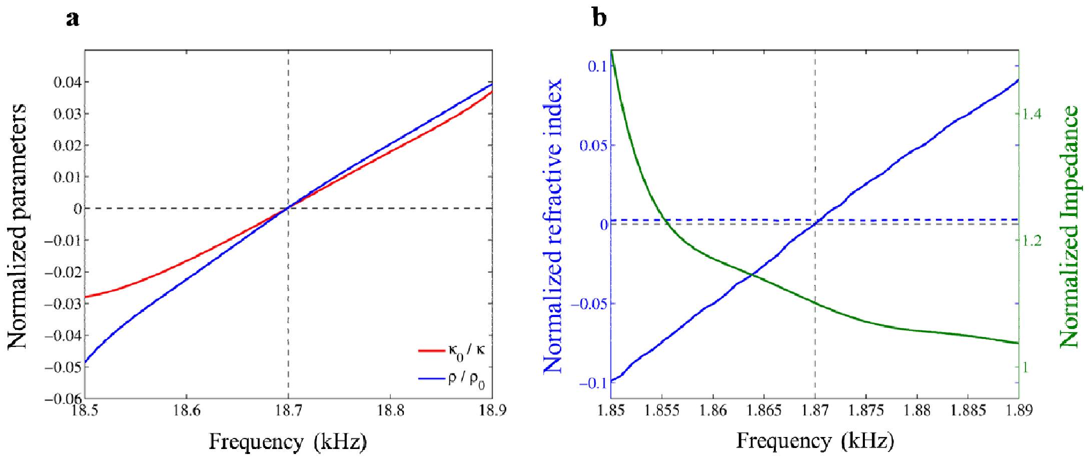

Supplementary Figure 2: Effective medium model of the double zero index metamaterial for the first order waveguide mode. (a) Normalized effective density and inverse bulk modulus. At 18.7 kHz, the effective density and inverse bulk modulus are simultaneously zero. (b) Normalized refractive index and impedance. The imaginary part of the refractive index is about 2.5×10^{-3} represented by the blue dashed line. At 18.7 kHz, the refractive index is zero and the impedance matches the background waveguide. Both (a) and (b) are sufficient to demonstrate double zero index properties.

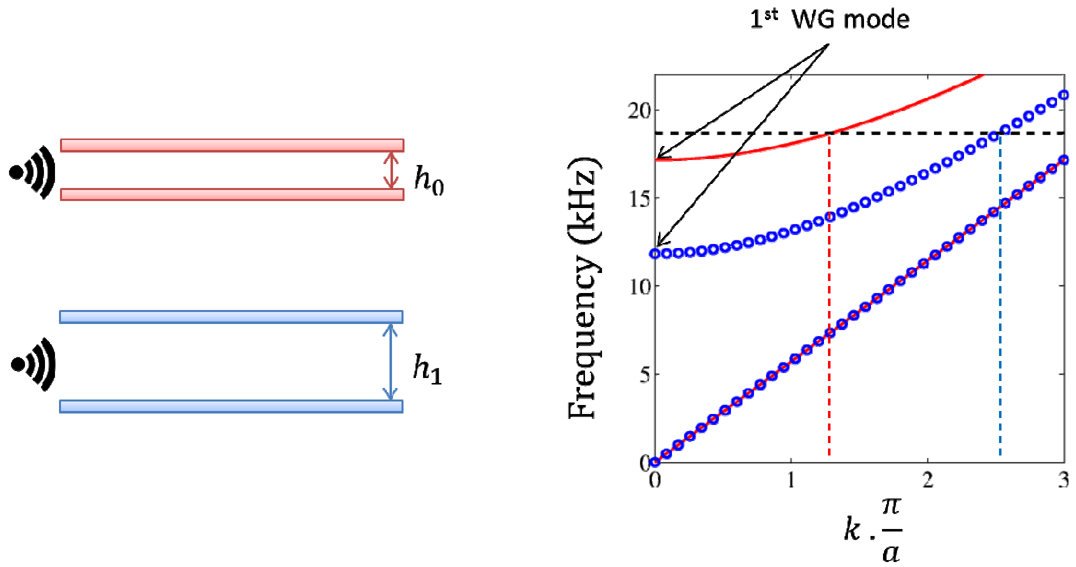

Supplementary Figure 3: Dispersion relation of the first order waveguide mode. The red and blue curves correspond to the thinner and thicker waveguides with air channel thickness $h_0 = 10$ mm and $h_1 = 14.5$ mm, respectively. At 18.7 kHz, the thinner waveguide has smaller wave vector, resulting in larger phase velocity. The ratio between the refractive indices, inversely proportional to the associated phase velocities, is $n_1/n_0 = 1.94$.

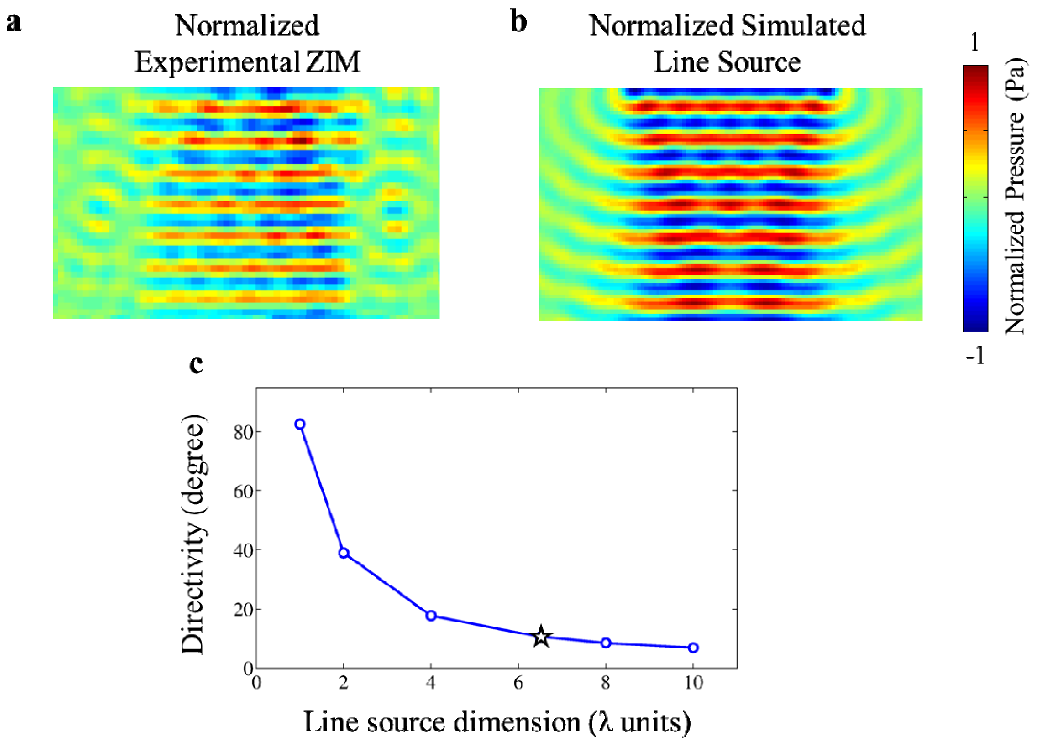

Supplementary Figure 4: Theoretical limits of directivity. (a, b) Normalized fields of plane waves generated by the zero index metamaterial in our experiment and a line source with the same size in simulation, respectively. The directivity of our experimental result shown in (a) is 11$\pm$1 degrees, close to the simulation of the line source shown in (b). (c) Theoretical limits of directivity with varying line source dimension. Line source with larger dimension generates plane waves with better directivity. The line source with length 6.5 wavelengths shown as the star in (c) corresponding to (b) has a limit 10.6 degrees.

Supplementary Note 1: Single zero index metamaterial

To investigate the difference between double and single zero index metamaterials, we detune the lattice constant of our designed structure to 2.7 cm that opens up the Dirac cone at the Brillouin zone centre (Supplementary Figures 1a, b), resulting in a single zero index metamaterial. The full wave simulations of one-dimensional waveguides with eight unit cells of single and double zero index metamaterials for normal incident first order waveguide mode plane wave at the zero index frequency f_0 are shown in Supplementary Figures 1c, d, respectively. The solid side walls act as periodic boundaries as discussed in the main text. The transmission through the single zero index metamaterial is much lower than the double zero index metamaterial. Supplementary Figure 1e shows the simulated (red) and measured (black) transmission through the double zero index metamaterial and the simulated (blue) transmission through the single zero index metamaterial. The transmission through the double zero index metamaterial has the same value discussed in the main text. At the zero index frequency f_0, a bandgap can be observed due to impedance mismatch, resulting in 30% transmission through the single zero index metamaterial.

Supplementary Note 2: Effective medium model

To further confirm that our designed metamaterial is a double zero index metamaterial, we retrieve the effective material parameters of a slab containing five unit cells by measuring the transmission and reflection in our full wave simulation with normal incidence [1]. The normalized effective density and bulk modulus are simultaneously zero at the Dirac point frequency, indicating that the metamaterial presents double zero index properties at this frequency (Supplementary Figure 2a). This characterization is equivalent to the model with refractive index and impedance. The normalized index is zero at 18.7 kHz, and the impedance matched with the surrounding waveguide ($Z = 1.1Z_0$ for simulation and $Z = 1.3Z_0$ for experiment shown in the main text, with Z_0 to be the impedance of the plain waveguide). The measured imaginary part of the refractive index is about 2.5×10^{-3} through the measured frequency range (blue dashed line in Supplementary Figure 2b), confirming that the metamaterial has zero real and imaginary part of the refractive index at 18.7 kHz.

Supplementary Note 3: The first order waveguide mode and its sound speed

The first order waveguide mode is acoustic wave propagating in a waveguide whose

maximum pressure amplitudes occur at the two boundaries of the waveguide and are phase opposite. Thus, the pressure distribution along the *z*-axis is anti-symmetric about the centre line of the waveguide. Such first order waveguide mode is used in all of our experiments because the designed zero refractive index material works for this acoustic propagating mode. The wave number along the *z*-axis is

$$k_z = \frac{\pi}{h}, \tag{1}$$

where h is the height of the waveguide. Based on the balance of linear momentum, the propagating wave number along *x*-axis is

$$k_x = \sqrt{k^2 - k_z^2} = \sqrt{\frac{\omega^2}{c_0^2} - \frac{\pi^2}{h^2}}, \tag{2}$$

where k is the total wave number, ω the angular frequency, and $c_0 = 343\ \mathrm{ms}^{-1}$ is the sound speed in air. Thus, the sound speed of the first order waveguide mode is

$$c = \frac{\omega}{k_x} = \left(\sqrt{\frac{1}{c_0^2} - \frac{\pi^2}{\omega^2 h^2}}\right)^{-1}. \tag{3}$$

Supplementary Eq. (3) indicates that a waveguide with larger air channel thickness exhibits lower phase velocity. This result is confirmed by the dispersions of waveguides with different air channel thickness shown in Supplementary Figure 3. The red (blue) dispersion curve for the first order waveguide mode corresponds to the waveguide with thinner (thicker) air channel. At the same frequency, the red dispersion curve associated with thinner air channel has smaller wave vector, resulting in larger phase velocity. For waveguides with 10 mm and 14.5 mm, the ratio of refractive indices inversely proportional to phase velocity is $n_1/n_0 = 1.94$ at the Dirac point frequency (18.7 kHz).

Supplementary Note 4: Directivity

The directivity of a plane wave can be expressed as the angle over which the amplitude of the plane wave is confined. As discussed in the main text, the amplitude generated by our zero index metamaterial sample excited by a point source is confined within 11 ± 1 degrees (Supplementary Figure 4a). A line source with the same dimension (30 cm, i.e. 6.5 wavelengths) generating a plane wave at 18.7 kHz is simulated by COMSOL 3.5b and shown in Supplementary Figure 4b. The comparison between the experimental and simulated fields indicates that the directivity performance of the zero index metamaterial is close to the theoretical limit. The theoretical limits of directivity with different line source dimensions are calculated (Supplementary Figure 4c). The directivity performance improves as the length of the line source becomes larger because line source with small dimensions compared with the wavelength behaves like a point source. For a line source with length 30 cm, the theoretical limit of directivity is 10.6 degrees as shown by the star in Supplementary

Figure 4c. This result confirms that the directivity performance of our sample is neat the theoretical limit.

Supplementary References

[1] Fokin, V., Ambati, M., Sun, C., and Zhang, X., Method for retrieving effective properties of locally resonant acoustic metamaterials, *Phys. Rev. B* **76**, 14432 (2007).
