# Supplementary material for: Observation of acoustic Dirac-like cone and double zero refractive index
Source: Nat Commun. 2017 Mar 20;8:14871. doi: 10.1038/ncomms14871 (PMC5364384; doi:10.1038/ncomms14871)
Supplement: Peer Review File [file ncomms14871-s5.pdf]

Editorial Note: this manuscript has been previously reviewed at another journal that is not operating a transparent peer review scheme. This document only contains reviewer comments and rebuttal letters for versions considered at Nature Communications.
